# Polygenic risk score and risk of monoclonal B-cell lymphocytosis in caucasians and risk of chronic lymphocytic leukemia (CLL) in African Americans

**DOI:** 10.1038/s41375-021-01344-9

**Published:** 2021-07-20

**Authors:** Geffen Kleinstern, J. Brice Weinberg, Sameer A. Parikh, Esteban Braggio, Sara J. Achenbach, Dennis P. Robinson, Aaron D. Norman, Kari G. Rabe, Nicholas J. Boddicker, Celine M. Vachon, Connie E. Lesnick, Timothy G. Call, Danielle M. Brander, Laura Z. Rassenti, Thomas J. Kipps, Janet E. Olson, James R. Cerhan, Neil E. Kay, Richard R. Furman, Curtis A. Hanson, Tait D. Shanafelt, Susan L. Slager

**Affiliations:** 1 School of Public Health, University of Haifa, Haifa, Israel; 2 Department of Quantitative Health Sciences, Mayo Clinic, Rochester, MN USA; 3 Department of Medicine, Duke University, Duke Cancer Institute, Durham, NC USA; 4 Department of Immunology, Duke University Medical Center, Durham, NC USA; 5 Durham Veterans Affairs Medical Center, Durham, NC USA; 6 Division of Hematology, Mayo Clinic, Rochester, MN USA; 7 Department of Hematology and Oncology, Mayo Clinic, Phoenix, AZ USA; 8 Moores Cancer Center, University of California, San Diego, San Diego, CA USA; 9 Weill Cornell Medical College/New York Presbyterian Hospital, New York, NY USA; 10 Department of Laboratory Medicine and Pathology, Mayo Clinic, Rochester, MN USA; 11 Department of Medicine, Division of Hematology, Stanford University, Stanford, CA USA

**Keywords:** Risk factors, Epidemiology, Genetics research

## Abstract

Monoclonal B-cell lymphocytosis (MBL) is a precursor to CLL. Other than age, sex, and CLL family-history, little is known about factors associated with MBL risk. A polygenic-risk-score (PRS) of 41 CLL-susceptibility variants has been found to be associated with CLL risk among individuals of European-ancestry(EA). Here, we evaluate these variants, the PRS, and environmental factors for MBL risk. We also evaluate these variants and the CLL-PRS among African-American (AA) and EA-CLL cases and controls. Our study included 560 EA MBLs, 869 CLLs (696 EA/173 AA), and 2866 controls (2631 EA/235 AA). We used logistic regression, adjusting for age and sex, to estimate odds ratios (OR) and 95% confidence intervals within each race. We found significant associations with MBL risk among 21 of 41 variants and with the CLL-PRS (OR = 1.86, *P* = 1.9 × 10^−29^, c-statistic = 0.72). Little evidence of any association between MBL risk and environmental factors was observed. We observed significant associations of the CLL-PRS with EA-CLL risk (OR = 2.53, *P* = 4.0 × 10^−63^, c-statistic = 0.77) and AA-CLL risk (OR = 1.76, *P* = 5.1 × 10^−5^, c-statistic = 0.62). Inherited genetic factors and not environmental are associated with MBL risk. In particular, the CLL-PRS is a strong predictor for both risk of MBL and EA-CLL, but less so for AA-CLL supporting the need for further work in this population.

## Introduction

Chronic lymphocytic leukemia (CLL) is a neoplasm of mature B-cells, with at least 5 × 10^9^ B-cells/L in the peripheral blood [1]. These CLL cells typically co-express CD5, CD19, CD20^dim^ and CD23, and exhibit a decrease in expression of surface immunoglobulin, CD20, and CD79b as compared to normal B cells [2, 3]. Leukemic B-cells also show restricted expression of either kappa or lambda immunoglobulin light chains featuring the clonal nature of such cells [2].

Monoclonal B-cell lymphocytosis (MBL) is a pre-malignant condition with a clonal absolute B-cell count of <5 × 10^9^/L in the peripheral blood, with the notable absence of lymphadenopathy, cytopenias, or organomegaly [1], and an immunophenotype that is similar to that of CLL. MBL is a precursor state to CLL [4, 5]. MBL clones are present in ~5–12% in the general population [6–8] with the prevalence rising to 15–22% in unaffected first- degree relatives of CLL patients [4, 9, 10]. MBL is also sub-classified into low-count MBL (LC-MBL) or high-count MBL (HC-MBL) according to the B-cell clone size of below or above 0.5 × 10^9^/L threshold, respectively [4, 6, 11]. Other than age, sex, and family history of CLL, little is known about factors associated with risk of MBL.

To date, 41 single nucleotide polymorphisms (SNPs) have been found to be associated with risk of CLL among European ancestry (EA) individuals, and they explain ~25% of the additive heritable risk [12–19]. We previously showed that a PRS of the weighted average of the number of risk alleles of these 41 SNPs is associated with CLL risk using cases and controls of EA from the International Lymphoma Epidemiology (InterLymph) Consortium [20]. However, these InterLymph cases and controls were used to identify over half of the SNPs, potentially inflating the association. Thus, we evaluated this CLL-PRS in an independent sample of CLL cases and controls from the Genetic Epidemiology of CLL (GEC) Consortium, a cohort of families each with ≥2 members with CLL. This analysis demonstrated that CLL-PRS along with age and sex has high discrimination (c-statistic = 0.78) for CLL risk. In these CLL families, we also reported an association of these 41 SNPs and the CLL-PRS with MBL risk in a small cohort of 95 familial MBLs; the vast majority (93%) of these were LC-MBL [20].

Here we evaluate these 41 SNPs, the CLL-PRS, and environmental factors in a large screening cohort of 560 EA MBLs (including 396 LC-MBLs and 164 HC-MBLs) and 2631 EA controls known not to have MBL, all of whom ascertained agnostic to family history status. Because the CLL-PRS has not been evaluated in non-EA individuals, particularly in African Americans (AA), we also evaluate the CLL-PRS in 173 AA CLL cases and 235 AA controls and compare these results to another independent cohort of 696 EA CLLs.

## Methods

### Study population

#### MBL and control individuals

To identify individuals with MBL, we had two EA cohorts: a screening cohort and a clinical cohort (Supplementary Fig. 1). For the screening cohort, we used stored cryopreserved peripheral blood mononuclear cells (PBMC) from 3041 asymptomatic adults participating in the Mayo Clinic Biobank to screen for MBL using a highly sensitive flow cytometry. Each consented participant in the Mayo Clinic Biobank was asked to complete a self-reported health-history questionnaire, provide a blood sample, and allow access to their Mayo Clinic medical record [21]. The baseline health-history questionnaire was a self-reported questionnaire that included domains around medical history, lifestyle factors, family history of hematological malignancies (any non-Hodgkin lymphoma, Hodgkin lymphoma, multiple myeloma, or leukemia), reproductive history, and occupational exposures [21] (Supplementary Table 1). We screened for MBL using a highly sensitive, 8-color (CD38, CD45, Kappa, Lambda, CD19, CD23, CD5 and CD20) flow-cytometry assay with the capacity to detect clonal B-cell counts to the 0.005% level (1/20,000 events), and for each individual, 500,000 PBMC events were typically captured [22]. Based on our MBL screening, we identified 410 individuals with CLL phenotype MBL (i.e., CD5^+^ CD20^dim^), with the remaining 2631 individuals without MBL serving as controls. Because the Mayo Clinic biobank participants did not all have a complete blood count, we used the percent of clonal B-cells out of total B-cells to categorize participants as LC- and HC-MBL [4]. Based on prior evidence, those MBL individuals with a percent clonal B-cell <85% were defined as LC-MBL and those with percent clonal B-cells ≥85% as HC-MBL [4]. Our second MBL cohort is a clinical cohort of predomominantly (99%) HC-MBL from the Mayo Clinic CLL Resource. This resource is comprised of individuals with a clonal B-cell population of CLL immunophenotype who are seen on a routine basis for clinical evaluations in the Division of Hematology at Mayo Clinic (Rochester, MN). All diagnoses were confirmed by a Mayo hematopathologist based on the 1996 NCI working group criteria and then updated to the 2008 International Workshop CLL criteria. From this CLL Resource, we identified 150 MBLs who had available DNA collected within 2 years of the initial MBL diagnosis. MBL was classified by LC-MBL or HC-MBL according to the B-cell clone size of below or above 0.5 × 10^9^/L threshold, respectively [6, 11] (Supplementary Fig. 1).

#### CLL patients and control individuals

CLL patients of EA or AA were ascertained from four studies (Supplementary Fig. 1): Mayo Clinic, Duke University, Weill Cornell Medical College, and the CLL Research Consortium (CRC). We identified 433 CLL patients (417 EA, 16 AA) from the Mayo Clinic CLL Resource who were diagnosed between 2002 and 2019 and who had available DNA collected within 2 years of CLL diagnosis. From Duke University, a total of 338 CLL patients (258 EA, 80 AA) were accrued from the CLL Clinic from 1999 through 2019 [23, 24]. From the CLL Research Consortium (CRC), we included 71 CLL patients (67 AA and 4 EA) [25]. Finally, from Weill Cornell Medical College, we included 27 CLL patients (17 EA, 10 AA) (Supplementary Fig. 1). CLL diagnoses were made based on the 1996 NCI working group criteria and updated to the 2008 International Workshop CLL criteria wherever possible. AA controls (*N* = 235) with no history of CLL were identified from the Mayo Clinic Biobank (Supplementary Fig. 1).

All individuals provided written informed consent approved by the respective institutional review board.

### Genotyping

Genotyping of the study cohort was done using Illumina genotyping arrays and genotypes were called using Illumina GenomeStudio software. Extensive quality control metrics were utilized including removing monomorphic SNPs, SNPs with call rates <95%, or SNPs with extreme Hardy–Weinberg disequilibrium (*P* < 1.0 × 10^−5^). We also dropped individuals with call rates <90%, gender discordance, or those who had a relative genotyped. Duplicates showed >99% concordance. From these data, we pulled the 41 SNPs previously found to be associated with CLL (Supplementary Tables 2 and 3). Using ADMIXTURE [26], we determined genetic ancestry for each individual using the HapMAP as the reference. Individuals with percent of African ancestry ≥50% were considered AA, and individuals with >80% Caucasian ancestry were considered EA. We correlated MAF between EA and AA across the 41 CLL SNPs using the 1000 genomes project data [27].

### Statistical analyses

We evaluated differences in the distribution of demographic characteristics and self-reported environmental exposures (including medical, lifestyle, family history, and occupational exposures) between cases and controls, using two-sided χ^2^ test or Student’s *t* test, where appropriate. Logistic regression was used to estimate OR and 95% confidence intervals (CIs), adjusted for age and sex. We computed the CLL-PRS based on the 41 CLL SNPs (Supplementary Tables 2 and 3) as previously published [20]. Specifically, the PRS was computed as a weighted average of the number of risk alleles across the 41 CLL SNPs, with the weights being the log of the odds ratio (OR) previously reported for each SNP (Supplementary Tables 2 and 3) [20]. We evaluated the CLL-PRS as a continuous or categorical predictor. Among EA analyses, we categorized the PRS by quintiles based on cutoffs previously used with 7983 controls from the InterLymph Consortium [20]. We also calculated an unweighted CLL-PRS and evaluated this unweighted PRS with CLL risk. For the AA analyses, we categorized the PRS quintiles based on 235 AA controls obtained from this study and used the same weights as that in EA analyses; an unweighted CLL-PRS was also evaluated. We used logistic regression, adjusted for age and sex, to evaluate associations of the PRS with risk of CLL, MBL, or MBL subtypes, stratified by race. The middle quintile served as the reference category. Among EA individuals, we calculated a trend test among LC-MBL, HC-MBL, and CLL risk using the *P* value for heterogeneity from a polytomous logistic regression analysis. Moreover, we plotted a boxplot for the PRS among controls, LC-MBL, HC-MBL, and EA CLL, and evaluated the statistical difference using the Kruskal–Wallis test. To evaluate model discriminatory ability, we computed a *c*-statistic and 95% CIs [28] for the adjusted regression models. Two-sided *P* values < 0.05 indicated statistical significance. In addition to the PRS, we evaluated each of the 41 CLL SNPs with the risk of MBL overall, LC-MBL, HC-MBL, EA CLL, and AA CLL assuming a log additive model in logistic regression. Because these SNPs were selected a priori, we used the nominal level (*P* < 0.05) for statistical significance. The data were analyzed using Software Package for Statistics and Simulation (IBM SPSS version 25, IBM Corp, Armonk, NY, USA), and R 3.6.3 (R Foundation for Statistical Computing, Vienna, Austria).

## Results

We evaluated associations of the individual SNPs and the CLL-PRS in 3887 individuals of EA and 408 AA individuals. Collectively, this included 560 EA MBLs (396 LC-MBLs and 164 HC-MBLs), 696 EA CLLs, 173 AA CLLs, 2631 EA controls, and 235 AA controls. The demographics of these individuals are shown in Table 1.

**Table 1:** Demographic characteristics by phenotype.

Characteristics	European ancestry										African Americans			
	Controls		MBL overall		LC-MBL		HC-MBL		CLL		Controls		CLL	
	*N* = 2631		*N* = 560		*N* = 396		*N* = 164		*N* = 696		*N* = 235		*N* = 173	
	*N*	%	*N*	%	*N*	%	*N*	%	*N*	%	*N*	%	*N*	%
Gender														
Male	1030	39%	319	57%	217	55%	102	62%	479	69%	160	68.1%	113	64.9%
Age (years)														
Median (range)	64	(29–101)	70	(43–97)	72	(44–95)	68	(43–97)	62	(30–94)	61	(40–90)	59	(26–94)
Cohort														
Mayo Clinic Biobank	2631	100%	410	73.2%	394	99.5%	16	9.8%			235	100%		
Mayo Clinic CLL Resource			150	26.8%	2	0.5%	148	90.2%	417	59.9%			16	9.2%
Duke University									258	37.1%			80	46.2%
Cornell									17	2.4%			10	5.8%
CLL Research Consortium									4	0.6%			67	38.8%

*CLL* Chronic lymphocytic leukemia, *HC* high-count, *LC* low-count, *MBL* Monoclonal B-cell lymphocytosis.

### Individual CLL-susceptibility SNPs and risk of CLL and MBL

The results for each of the 41 individual CLL-susceptibility SNPs for risk of CLL, MBL and MBL subtypes (LC-MBL and HC-MBL) are shown in Supplementary Tables 2 and 3. Among CLL cases and controls of EA, the ORs of 40 (98%) SNPs out of the 41 were directionally consistent with those reported in the larger CLL GWAS studies [12–19], and 32 (78%) SNPs out of the 41 were statistically significant at *P* < 0.05 (Supplementary Table 3). We also evaluated the 41 individual CLL-susceptibility SNPs among AA CLL cases and controls (Supplementary Table 3). Among the 41 SNPs, ORs of 22 SNPs (54%) were directionally consistent with those reported in CLL GWAS of EA and only two SNPs, rs7690934 (OR = 1.41, CI: 1.03–1.95, *P* = 0.03) and rs1679013 (OR = 1.56, CI: 1.08–2.25, *P* = 0.02), were nominally significant (Supplementary Table 3). The lack of statistical significance for the other SNPs in the AA appears to be due in part to the variability of minor allele frequencies (MAF) across EA and AA. The median difference in the MAF between EA and AA across the 41 CLL SNPs was 7.2% (range: 0.2–26%) in the 1000 genomes, with the majority of the MAF in the AA being lower than that of EA (Supplementary Table 4, Supplementary Fig. 2). The lower MAF in the AA then translates to attenuated ORs in the AA compared to that in the EA (Supplementary Fig. 3). Among MBL overall, the observed ORs for 39 (95%) of the 41 SNPs were directionally consistent with those reported in CLL, and 21 (51%) of the 41 SNPs were nominally statistically significant at *P* < 0.05 and 15 of the 41 SNPs showed little evidence of an association (OR < 1.1) (Supplementary Table 2).

### CLL-PRS and risk of MBL overall

The median CLL-PRS was 7.90 and 7.46 among 560 MBLs and 2631 controls of EA, respectively (Table 2). The PRS distribution among controls was consistent and overlapped with the distribution of 7983 controls from the InterLymph Consortium [20] (Supplementary Fig. 4). The continuous PRS had a 1.86-fold increased risk for MBL (CI: 1.67–2.07, *P* = 1.9 × 10^−29^), with a c-statistic of 0.72 (CI: 0.69–0.73) (Table 2). Compared to the middle quintile, the highest quintile had 2.38-fold increased risk for MBL (CI: 1.81–3.13, *P* = 5.5 × 10^−10^), and the lowest quintile had a 54% reduced risk (OR = 0.46, CI: 0.32–0.66, *P* = 2.9 × 10^−5^) (Table 2). The 99th percentile (5.5% of MBL) compared to the middle quintile had a 4.83-fold increased risk for MBL (CI: 2.81–8.31, *P* = 1.3 × 10^−8^).

**Table 2:** PRS and association with MBL risk among individuals of European ancestry.

PRS^b^	Controls^c^		MBL		MBL vs controls		
	*N* = 2631		*N* = 560				
	*N*	%	*N*	%	OR^a^	95% CI	*P*
Q1 [4.32, 6.80)	597	22%	50	9%	0.46	0.32–0.66	2.9 × 10^−5^
Q2 [6.80, 7.32)	528	20%	90	16%	0.88	0.64–1.20	0.42
Q3 [7.32, 7.77)	553	21%	103	18%	1	Reference	
Q4 [7.77, 8.28)	463	18%	110	20%	1.32	0.97–1.79	0.07
Q5 [8.28, 11.31)	490	19%	207	37%	2.38	1.81–3.13	5.5 × 10^−10^
Continuous					1.86	1.67–2.07	1.9 × 10^−29^
Continuous unweighted					1.15	1.13–1.18	2.4 × 10^−31^
PRS (median)	7.46		7.90				
*c*-statistic					0.72	0.69–0.73	
*c*-statistic unweighted					0.72	0.70–0.74	

*CI* confidence interval, *MBL* Monoclonal B-cell lymphocytosis, *OR* odds ratio, *PRS* polygenic risk score, *Q* quintile.

^a^Adjusted for age and sex.

^b^Quintiles based on 7983 controls.

^c^These controls were screened negative for MBL.

### CLL-PRS and risk of LC-MBL

Among 396 LC-MBL, only 10% were in the lowest PRS quintile, while 34% were in the highest quintile. The median PRS was 7.84, and the continuous PRS had a 1.75-fold increased risk for LC-MBL (CI: 1.55–1.98, *P* = 7.5 × 10^−19^) compared to the Biobank controls, with a c-statistic of 0.72 (CI: 0.70–0.75) (Table 3). Compared to the middle quintile, the highest quintile had 2.10-fold increased risk for LC-MBL (CI: 1.53–2.88, *P* = 4.0 × 10^−6^), and the lowest quintile had a 49% reduced risk (OR = 0.51, CI: 0.34–0.76, *P* = 0.001) (Table 3). The 99th percentile (4.3% of LC-MBL) compared to the middle quintile had a 3.69-fold increased risk for LC-MBL (CI: 1.94–7.02, *P* = 6.8 × 10^−5^).

**Table 3:** PRS and association with MBL subtypes and CLL among individuals of European ancestry.

PRS^b^	Controls^c^		LC-MBL		HC-MBL		CLL		LC-MBL vs controls			HC-MBL vs Controls			CLL vs Controls		
	*N* = 2631		*N* = 396		*N* = 164		*N* = 696										
	*N*	%	*N*	%	*N*	%	*N*	%	OR^a^	95% CI	*P*	OR^a^	95% CI	*P*	OR^a^	95% CI	*P*
Q1 [4.32, 6.80)	597	22%	41	10%	9	6%	35	5%	0.51	0.34–0.76	0.001	0.33	0.15–0.70	0.004	0.31	0.21–0.46	1.0 × 10^−8^
Q2 [6.80, 7.32)	528	20%	62	16%	28	17%	71	10%	0.81	0.57–1.17	0.27	1.05	0.61–1.82	0.85	0.65	0.47–0.90	0.01
Q3 [7.32, 7.77)	553	21%	76	19%	27	16%	112	16%	1	Reference		1	Reference		1	Reference	
Q4 [7.77, 8.28)	463	18%	82	21%	28	17%	142	20%	1.33	0.95–1.88	0.1	1.31	0.76–2.26	0.34	1.56	1.17–2.08	0.002
Q5 [8.28, 11.31)	490	19%	135	34%	72	44%	336	49%	2.10	1.53–2.88	4.0 × 10^−6^	3.13	1.97–4.98	1.0 × 10^−6^	3.49	2.70–4.51	1.2 × 10^−21^
Continuous									1.75	1.55–1.98	7.5 × 10^−19^	2.14	1.80–2.56	3.9 × 10^−17^	2.53	2.27–2.81	4.0 × 10^−63^
Continuous unweighted									1.14	1.11–1.17	1.3 × 10^−20^	1.19	1.14–1.23	1.9 × 10^−17^	1.23	1.20–1.26	4.1 × 10^−65^
PRS (median)	7.46		7.84		8.05		8.24										
*c*-statistic									0.72	0.70–0.75		0.73	0.69–0.77		0.77	0.75–0.79	
*c*-statistic unweighted									0.72	0.70–0.75		0.725	0.69–0.77		0.775	0.76–0.79	

*CI* confidence interval, *CLL* Chronic lymphocytic leukemia, *HC* high-count, *LC* low-count, *MBL* Monoclonal B-cell lymphocytosis, *OR* odds ratio, *PRS* polygenic risk score, *Q* quintile.

^a^Adjusted for age and sex.

^b^Quintiles based on 7983 controls.

^c^These controls were screened negative for MBL.

### CLL-PRS and risk of HC-MBL

**Fig. 1:**
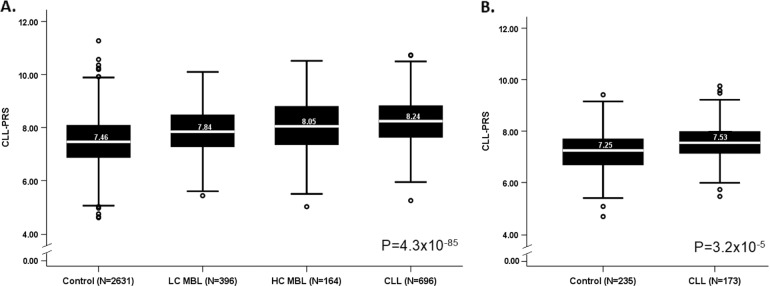
Polygenic risk score distribution among controls, LC-MBL, HC-MBL, and CLL European ancestry and African-American individuals. **A** Boxplots representing the CLL-PRS distribution among EA controls, LC-MBL, HC-MBL, and CLL. The white line in the box represents the median score of 7.46, 7.84, 8.05, and 8.24 for controls, LC-MBL, HC-MBL, and CLL, respectively. *P* value represents the statistical difference of the CLL-PRS between the four groups. **B** Boxplots representing the CLL-PRS distribution among AA controls and CLL. The white line in the box represents the median score of 7.25 and 7.53 for controls and CLL, respectively. *P* value represents the statistical difference between the CLL-PRS in AA controls and CLL. Y-axis (CLL-PRS) represents a weighted average across 41 CLL risk SNPs. AA African-American, CLL chronic lymphocytic leukemia, EA European Ancestry, HC high-count, LC low-count, MBL Monoclonal B-cell lymphocytosis, PRS polygenic risk score.

Among 164 HC-MBL individuals, only 6% were in the lowest PRS quintile, while 44% were in the highest quintile. The median PRS was 8.05 which was higher than the LC-MBL PRS (Fig. 1). When comparing the HC-MBL individuals to the 2631 Biobank controls, the continuous PRS had a 2.14-fold increased risk for HC-MBL (CI: 1.80–2.56, *P* = 3.9 × 10^−17^), with a c-statistic of 0.73 (CI: 0.69–0.77) (Table 3). Compared to the middle quintile, the highest quintile had 3.13-fold increased risk for HC-MBL (CI: 1.97–4.98, *P* = 1.0 × 10^−6^), and the lowest quintile had a 0.33-fold decreased risk (CI: 0.15–0.70, *P* = 0.004) (Table 3). The 99th percentile (8.5% of HC-MBL) compared to the middle quintile had an 8.18-fold increased risk for HC-MBL (CI: 3.85–17.4, *P* = 4.6 × 10^−8^).

### CLL-PRS and risk of CLL among individuals of EA

Among 696 CLL patients, only 5% were in the lowest PRS quintile, while 49% were in the highest quintile. The median PRS was 8.24 which was higher than both the LC-MBL and HC-MBL PRS (Fig. 1). When comparing the CLL cases to the Biobank controls, the continuous PRS had a 2.53-fold increased risk for CLL (CI: 2.27–2.81, *P* = 4.0 × 10^−63^), with a c-statistic of 0.77 (CI: 0.75–0.79) (Table 3). Compared to the middle quintile, the highest quintile had 3.49-fold increased risk for CLL (CI: 2.70–4.51, *P* = 1.2 × 10^−21^), and the lowest quintile had a 0.31-fold decreased risk (CI: 0.21–0.46, *P* = 1.0 × 10^−8^) (Table 3). The 99th percentile (6.6% of CLL) compared to the middle quintile had a 5.98-fold increased risk for CLL (CI: 3.61–9.93, *P* = 4.3 × 10^−12^).

When comparing the PRS between controls, LC-MBL, HC-MBL, and CLL, we found a significant difference (*P* = 4.3 × 10^−85^, Fig. 1A). There was also a significant positive trend between the PRS effect sizes and risk of LC-MBL, HC-MBL, and CLL, with the association increasing as the clonal size increases (*P*_heterogeneity_ = 1.5 × 10^−5^).

### CLL-PRS and risk of CLL among African-American individuals

We calculated the EA CLL-PRS among 173 AA CLL and 235 AA controls. The median PRS was 7.53 and 7.25 among CLL and controls, respectively (Table 4, Fig. 1B). We observed a 1.76-fold increased risk of CLL (CI: 1.34–2.31, *P* = 5.1 × 10^−5^) with a c-statistic of 0.62 (CI: 0.57–0.68). Moreover, when eliminating the weights that were generated from the EA, the unweighted CLL-PRS effect size attenuated but still statistically significant (continuous OR = 1.07, CI: 1.01–1.13, *P* = 0.03) (Table 4).

**Table 4:** Association between the CLL-PRS and risk of CLL among African Americans.

CLL-PRS^b^	Control		CLL		CLL vs controls		
	*N* = 235		*N* = 173				
	*N*	%	*N*	%	OR^a^	95% CI	*P*
Q1 [4.32,6.80)	47	20.0%	14	8.1%	0.47	0.22–0.99	0.048
Q2 [6.80,7.32)	47	20.0%	27	15.6%	0.88	0.45–1.70	0.70
Q3 [7.32,7.77)	46	19.6%	30	17.3%	1.00	Reference	
Q4 [7.77,8.28)	47	20.0%	50	28.9%	1.60	0.87–2.95	0.13
Q5 [8.28,11.31)	48	20.4%	52	30.1%	1.61	0.88–2.96	0.12
Continuous					1.76	1.34–2.31	5.1 × 10^−5^
Continuous unweighted					1.07	1.01–1.13	0.03
PRS (median)	7.25		7.53				
*c*-statistic weighted					0.62	0.57–0.68	
*c*-statistic unweighted					0.57	0.53–0.64	

*CI* confidence interval, *CLL* chronic lymphocytic leukemia, *OR* odds ratio, *PRS* polygenic risk score, *Q* quintile.

^a^Adjusted for age and sex.

^b^Quintiles based on 235 African-American controls distribution.

### Environmental exposures and risk of MBL in the Mayo Clinic Biobank

In the Mayo Clinic Biobank, we had 2512 controls and 365 MBL individuals who completed a self-reported questionnaire. Because the vast majority of MBLs from the Biobank were LC-MBL (only 9 individuals were HC-MBL), we evaluated the effect of these exposures on MBL risk overall (Supplementary Table 1). As expected, age per 10 years (OR = 1.83, CI: 1.64–2.04, *P* < 0.0001) and male sex (OR = 1.73, CI: 1.38–2.15, *P* < 0.0001) were associated with higher risk of MBL. Family history of leukemia/lymphoma was higher among MBL cases (*N* = 40, 13.1%) compared to controls (*N* = 205, 9.5%); however, it did not cross the threshold of significance (OR = 1.44, CI: 0.99–2.09, *P* = 0.06, adjusted for age and sex, Supplementary Table 1). Prior history of cancer other than leukemia or lymphoma was significantly higher (*P* < 0.0001) among MBL cases (*N* = 156, 43%) compared to controls (*N* = 769, 31%); however, the association was not statistically significant after adjusting for age and sex (OR = 1.22, CI: 0.96–1.55, *P* = 0.10, Supplementary Table 1). Within specific cancers, prior history of melanoma and non-melanoma skin cancers, prior history of sarcoma, and, among women, prior history of breast cancer were significantly higher in MBL cases compared to controls; however, none of these specific prior cancers were associated with MBL risk after adjusting for age and sex (Supplementary Table 1). No other exposures were found to be statistically associated with MBL risk, including prior history of type 2 diabetes, prior history of any autoimmune condition, or prior diagnosis of hepatitis A, B, or C.

## Discussion

Our study clearly demonstrated that an inherited genetic component exists for the development of MBL, both among a cohort of 410 asymptomatic individuals from the Mayo Clinic Biobank who were screened for MBL and among a cohort of 150 MBLs who were clinically identified in the Division of Hematology. We observed that ~50% of the known 41 SNPs from 37 CLL-susceptibility loci and the CLL-PRS comprised of these 41 SNPs were associated with MBL overall risk. Two prior studies evaluated risk of MBL with SNPs from 10 CLL-susceptibility loci among 419 MBLs [29] and from 8 CLL-susceptibility loci among 60 familial MBLs from CLL families [30]. All three studies found statistically significant associations with SNPs in the 2q37.1 locus, and two of the three studies (excluding the familial MBLs) found significant associations at the 6p25.3, 8q24.21, 11q24.1 and 16q24.1 loci. Our study also found associations at these loci. With the additional 32 CLL-susceptibility loci evaluated herein, we found significant SNP associations with MBL risk from 12 more loci. Of particular interest, we found no or limited evidence of association (OR < 1.10 and *P* > 0.05) for 12 known CLL risk loci. Because the SNPs in these loci have been repeatedly found to be associated with risk of CLL, this suggests that these loci may be associated with progression from MBL to CLL rather than associated with initiation of the B-cell clone. Further studies are needed to evaluate this hypothesis.

We previously reported that the CLL-PRS was associated with MBL risk among a cohort of 95 familial MBLs with a 2.3-fold increased risk [20]. Herein, among a cohort ascertained agnostic to family history of CLL, we also reported an association of the CLL-PRS with risk of MBL. In both studies, the CLL-PRS had good discrimination (after adjusting for age and sex) with an estimated c-statistic of 0.77 in the family study and 0.72 in this study. We next evaluated the CLL-PRS among the LC-MBL and HC-MBL subsets. We observed a significant association with a 1.75-fold and 2.14-fold increased risk for LC-MBL and HC-MBL, respectively. Moreover, the increase in the effect size from LC-MBL to HC-MBL to CLL was statistically significant. Because not all MBLs progress to CLL, the next needed study is to determine whether the CLL-PRS could discriminate progression to CLL among indivdiuals with MBL. Based on our data, there is strong evidence that those MBLs with high PRS will have a greater chance of progression to CLL compared to those MBLs with a low PRS.

For the first time, we evaluated the CLL-PRS in AA CLL cases and controls based on genetic ancestry and found a significant increased risk for CLL, though, with an attenuated effect (1.76-fold) and less discrimination (the c-statistic = 0.62) compared to our EA CLL cases and controls. These findings are not surprising given the known differences in the genetic landscape (i.e., allele frequencies and linkage disequilibrium) between populations of EA and AA. Moreover, the CLL-PRS is comprised of SNPs that were identified through GWAS of individuals with EA ancestry and includes the estimated ORs from these EA GWAS as the weights in the PRS calculation instead of weights obtained from AA GWAS of CLL, which has yet to be done. When we used an unweighted PRS, we also observed a significant, although attenuated, association. These results highlight that the EA PRS is a weak predictor for AA individuals compared to EA individuals. Thus there is a need for a GWAS of CLL among AA in order to identify CLL-susceptibility SNPs which may be unique to AA CLL or SNPs that are more informative within known CLL loci. A PRS can then be developed based on these more representative SNPs.

We previously reported that the CLL-PRS had a 2.49-fold increased risk among CLL cases and controls of EA from the InterLymph Consortium [20], but because these individuals were used to identify at least 50% of the CLL-susceptibility SNPs, the statistical significance and the effect size of the PRS would have been inflated (i.e., winner’s curse [31]). Thus, we used an independent cohort of EA CLL cases and controls and reported consistent results of the CLL-PRS with a 2.53-fold increased risk. We also previously evaluated the CLL-PRS among CLL cases and control ascertained from CLL families that had at least 2 family members with CLL and also found consistent effect of the CLL-PRS with a 2.44-fold increased risk [20]. Importantly, across these three sets of CLL cases and controls, we also see strong and consistent discriminatory ability of the CLL-PRS, along with age and sex, with c-statistics of 0.79, 0.80, and 0.77, respectively. Collectively, these results affirm and again demonstrate that the CLL-PRS is a strong predictor of CLL risk.

Among the environmental exposures evaluated beyond age and sex, we observed suggestive although not-significant after adjusting for age and sex that a prior history of cancer or a family history of leukemia or lymphoma may be associated with MBL risk. A prior study by Casabonne et al. of 72 MBLs and 380 controls screened not to have MBL also found suggestive evidence albeit not-significant that a prior history of cancers increased risk of MBL [32]. In addition, several family studies reported elevated prevalence rates of MBL among relatives of CLL families compared to that of the general population [9, 10, 33]. Casabonne et al. also found evidence that exposures to infectious agents (e.g., history of pneumonia) increased MBL risk and that prior history of vaccination (e.g., vaccinated against pneumococcal or influenza) decreased MBL risk [32]. No other medical, occupational, or lifestyle exposures evaluated herein were found to be associated with risk of MBL.

In conclusion, inherited genetic factors and not environmental are associated with risk of MBL. We reported that some, but not all of the CLL-susceptibility SNPs, and the CLL-PRS were associated with risk of initiation of the MBL clone among individuals of EA suggesting the possibility that the remaining SNPs are associated with progression to CLL. We also demonstrated that the CLL-PRS is a strong and significant predictor of risk for CLL among individuals of EA agnostic to family history and a somewhat weaker predictor of risk among AA individuals supporting the need for further work in this population. Most importantly the results of this study may help identify individuals at higher risk of developing MBL and CLL beyond the known risk associated with age, male sex, and family history of CLL in individuals of EA.

## Supplementary information

Supplemental Material

## References

[1] Hallek M, Cheson BD, Catovsky D, Caligaris-Cappio F, Dighiero G, Dohner H (2008). Guidelines for the diagnosis and treatment of chronic lymphocytic leukemia: a report from the International Workshop on Chronic Lymphocytic Leukemia updating the National Cancer Institute-Working Group 1996 guidelines. Blood.

[2] Moreau EJ, Matutes E, A’Hern RP, Morilla AM, Morilla RM, Owusu-Ankomah KA (1997). Improvement of the chronic lymphocytic leukemia scoring system with the monoclonal antibody SN8 (CD79b). Am J Clin Pathol.

[3] Ginaldi L, De Martinis M, Matutes E, Farahat N, Morilla R, Catovsky D (1998). Levels of expression of CD19 and CD20 in chronic B cell leukaemias. J Clin Pathol.

[4] Slager SL, Lanasa MC, Marti GE, Achenbach SJ, Camp NJ, Abbasi F, et al. Natural history of monoclonal B-cell lymphocytosis (MBL) among relatives in chronic lymphocytic leukemia (CLL) families. *Blood* 2021;137:2046–56. 10.1182/blood.2020006322 PMC8057266 33512457

[5] Landgren O, Albitar M, Ma W, Abbasi F, Hayes RB, Ghia P (2009). B-cell clones as early markers for chronic lymphocytic leukemia. N Engl J Med.

[6] Dagklis A, Fazi C, Sala C, Cantarelli V, Scielzo C, Massacane R (2009). The immunoglobulin gene repertoire of low-count chronic lymphocytic leukemia (CLL)-like monoclonal B lymphocytosis is different from CLL: diagnostic implications for clinical monitoring. Blood.

[7] Ghia P, Prato G, Scielzo C, Stella S, Geuna M, Guida G (2004). Monoclonal CD5+ and CD5- B-lymphocyte expansions are frequent in the peripheral blood of the elderly. Blood.

[8] Rawstron AC, Kennedy B, Evans PA, Davies FE, Richards SJ, Haynes AP (2001). Quantitation of minimal disease levels in chronic lymphocytic leukemia using a sensitive flow cytometric assay improves the prediction of outcome and can be used to optimize therapy. Blood.

[9] Goldin LR, Lanasa MC, Slager SL, Cerhan JR, Vachon CM, Strom SS (2010). Common occurrence of monoclonal B-cell lymphocytosis among members of high-risk CLL families. Br J Haematol.

[10] Marti GE, Carter P, Abbasi F, Washington GC, Jain N, Zenger VE (2003). B-cell monoclonal lymphocytosis and B-cell abnormalities in the setting of familial B-cell chronic lymphocytic leukemia. Cytom Part B Clin Cytom.

[11] Rawstron AC, Shanafelt T, Lanasa MC, Landgren O, Hanson C, Orfao A (2010). Different biology and clinical outcome according to the absolute numbers of clonal B-cells in monoclonal B-cell lymphocytosis (MBL). Cytom Part B Clin Cytom.

[12] Crowther-Swanepoel D, Broderick P, Di Bernardo MC, Dobbins SE, Torres M, Mansouri M (2010). Common variants at 2q37.3, 8q24.21, 15q21.3 and 16q24.1 influence chronic lymphocytic leukemia risk. Nat Genet.

[13] Di Bernardo MC, Crowther-Swanepoel D, Broderick P, Webb E, Sellick G, Wild R (2008). A genome-wide association study identifies six susceptibility loci for chronic lymphocytic leukemia. Nat Genet.

[14] Berndt SI, Skibola CF, Joseph V, Camp NJ, Nieters A, Wang Z (2013). Genome-wide association study identifies multiple risk loci for chronic lymphocytic leukemia. Nat Genet.

[15] Sava GP, Speedy HE, Di Bernardo MC, Dyer MJS, Holroyd A, Sunter NJ (2014). Common variation at 12q24.13 (OAS3) influences chronic lymphocytic leukemia risk. Leukemia.

[16] Speedy HE, Di Bernardo MC, Sava GP, Dyer MJS, Holroyd A, Wang Y (2014). A genome-wide association study identifies multiple susceptibility loci for chronic lymphocytic leukemia. Nat Genet.

[17] Berndt SI, Camp NJ, Skibola CF, Vijai J, Wang Z, Gu J (2016). Meta-analysis of genome-wide association studies discovers multiple loci for chronic lymphocytic leukemia. Nat Commun.

[18] Law PJ, Berndt SI, Speedy HE, Camp NJ, Sava GP, Skibola CF (2017). Genome-wide association analysis implicates dysregulation of immunity genes in chronic lymphocytic leukaemia. Nat Commun.

[19] Slager SL, Rabe KG, Achenbach SJ, Vachon CM, Goldin LR, Strom SS (2011). Genome-wide association study identifies a novel susceptibility locus at 6p21.3 among familial CLL. Blood.

[20] Kleinstern G, Camp NJ, Goldin LR, Vachon CM, Vajdic CM, de Sanjose S (2018). Association of polygenic risk score with the risk of chronic lymphocytic leukemia and monoclonal B-cell lymphocytosis. Blood.

[21] Olson JE, Ryu E, Johnson KJ, Koenig BA, Maschke KJ, Morrisette JA (2013). The Mayo Clinic Biobank: a building block for individualized medicine. Mayo Clin Proc.

[22] Shanafelt TD, Kay NE, Parikh SA, Achenbach SJ, Lesnick CE, Hanson CA, et al. Risk of serious infection among individuals with and without low count monoclonal B-cell lymphocytosis (MBL). *Leukemia* 2021;35:239–44. 10.1038/s41375-020-0799-8 PMC7501139 32203143

[23] Christensen DJ, Chen Y, Oddo J, Matta KM, Neil J, Davis ED (2011). SET oncoprotein overexpression in B-cell chronic lymphocytic leukemia and non-Hodgkin lymphoma: A predictor of aggressive disease and a new treatment target. Blood.

[24] Lanasa MC, Allgood SD, Volkheimer AD, Gockerman JP, Whitesides JF, Goodman BK (2010). Single-cell analysis reveals oligoclonality among ‘low-count’ monoclonal B-cell lymphocytosis. Leukemia.

[25] Coombs CC, Rassenti LZ, Falchi L, Slager SL, Strom SS, Ferrajoli A (2012). Single nucleotide polymorphisms and inherited risk of chronic lymphocytic leukemia among African Americans. Blood.

[26] Alexander DH, Novembre J, Lange K (2009). Fast model-based estimation of ancestry in unrelated individuals. Genome Res.

[27] Auton A, Abecasis GR, Altshuler DM, Durbin RM, Bentley DR, Chakravarti A (2015). A global reference for human genetic variation. Nature.

[28] Steyerberg EW, Vickers AJ, Cook NR, Gerds T, Gonen M, Obuchowski N (2010). Assessing the performance of prediction models: a framework for traditional and novel measures. Epidemiol.

[29] Crowther-Swanepoel D, Corre T, Lloyd A, Gaidano G, Olver B, Bennett FL (2010). Inherited genetic susceptibility to monoclonal B-cell lymphocytosis. Blood.

[30] Slager SL, Rabe KG, Achenbach SJ, Vachon CM, Goldin LR, Strom SS (2011). Genome-wide association study identifies a novel susceptibility locus at 6p21.3 among familial CLL. Blood.

[31] Palmer C, Pe’er I (2017). Statistical correction of the Winner’s Curse explains replication variability in quantitative trait genome-wide association studies. PLOS Genet.

[32] Casabonne D, Almeida J, Nieto WG, Romero A, Fernández-Navarro P, Rodriguez-Caballero A (2012). Common infectious agents and monoclonal B-cell lymphocytosis: a cross-sectional epidemiological study among healthy adults. PloS one.

[33] Matos DM, Ismael SJ, Scrideli CA, de Oliveira FM, Rego EM, Falcão RP (2009). Monoclonal B-cell lymphocytosis in first-degree relatives of patients with sporadic (non-familial) chronic lymphocytic leukaemia. Br J Haematol.

