# Supplementary material for: Polygenic risk score and risk of monoclonal B-cell lymphocytosis in caucasians and risk of chronic lymphocytic leukemia (CLL) in African Americans
Source: Leukemia. 2021 Jul 20;36(1):119–25. doi: 10.1038/s41375-021-01344-9 (PMC8727288; doi:10.1038/s41375-021-01344-9)
Supplement: Supplementary file 1 — Supplemental Material [file 41375_2021_1344_MOESM1_ESM.docx]

**Supplemental Table 1**: Association between environmental exposures and MBL risk

| **Type of exposure** | **Exposure** | **# Controls** | **# MBL** | **OR*** | **95% CI** | | **p-value** |
| --- | --- | --- | --- | --- | --- | --- | --- |
| Demographic characteristics | Age at sample per 10 years | 2512 | 365 | 1.826 | 1.635 | 2.040 | <.0001 |
|  | Male | 2512 | 365 | 1.726 | 1.384 | 2.152 | <.0001 |
|  | Race: White | 2501 | 363 | 1.091 | 0.608 | 1.957 | 0.7712 |
|  | Hispanic or Latino | 2495 | 364 | . | . | . | 0.9765 |
| Lifestyle exposures | Used any tobacco products for 12+ months | 2448 | 358 | 0.798 | 0.531 | 1.199 | 0.2767 |
|  | Ever used cigar for 12+ months | 2456 | 356 | 0.659 | 0.365 | 1.189 | 0.1658 |
|  | Ever used pipe for 12+ months | 2451 | 357 | 0.820 | 0.493 | 1.365 | 0.4454 |
|  | Ever used snuff for 12+ months | 2453 | 356 | 0.820 | 0.310 | 2.168 | 0.6897 |
|  | Ever used chewing tobacco for 12+ months | 2445 | 355 | 0.800 | 0.327 | 1.957 | 0.6244 |
|  | Smoked >=100 cigarettes in entire life | 2478 | 360 | 0.923 | 0.731 | 1.165 | 0.5005 |
|  | Alcohol drinks in past 12 months: <=1/mo vs >1mo | 2498 | 364 | 0.945 | 0.750 | 1.192 | 0.6351 |
| Occupational exposures | Ever regularly exposed to asbestos | 2200 | 306 | 0.652 | 0.421 | 1.011 | 0.0562 |
|  | Ever regularly exposed to benzene or derivatives | 2220 | 318 | 0.988 | 0.554 | 1.762 | 0.9671 |
|  | Ever regularly exposed to chlorinated hydrocarbons | 2237 | 313 | 0.799 | 0.524 | 1.218 | 0.2978 |
|  | Ever regularly exposed to chromium | 2215 | 310 | 0.836 | 0.348 | 2.008 | 0.6893 |
|  | Ever regularly exposed to coal dust | 2402 | 340 | 0.774 | 0.400 | 1.497 | 0.4458 |
|  | Ever regularly exposed to nickel | 2310 | 323 | 0.829 | 0.286 | 2.405 | 0.7303 |
|  | Ever regularly exposed to radioactive substance | 2331 | 327 | 0.903 | 0.423 | 1.929 | 0.7929 |
|  | Ever regularly exposed to taconite | 2322 | 329 | 0.487 | 0.143 | 1.655 | 0.2490 |
|  | Ever regularly exposed to other substances | 681 | 89 | 1.342 | 0.596 | 3.023 | 0.4771 |
|  | Ever personally mixed/applied ferilizer to soil | 2500 | 364 | 0.951 | 0.750 | 1.205 | 0.6750 |
|  | Ever personally mixed/applied herbicides to kill weeds | 2500 | 364 | 0.987 | 0.778 | 1.252 | 0.9139 |
|  | Ever lived on a working farm | 2500 | 364 | 1.055 | 0.839 | 1.327 | 0.6462 |
|  | Primary Work: farming, forestry, fishing, hunting | 2108 | 306 | 1.107 | 0.739 | 1.659 | 0.6228 |
| Diet | Time/day eat high-fat foods | 2506 | 365 | 0.902 | 0.759 | 1.073 | 0.2445 |
|  | Servings of fruit per day | 2506 | 364 | 1.122 | 1.002 | 1.256 | 0.0470 |
|  | Servings of vegetables per day | 2509 | 365 | 1.048 | 0.929 | 1.182 | 0.4469 |
|  | Servings of milk/dairy/calcium supplements per day | 2504 | 364 | 1.014 | 0.837 | 1.227 | 0.8905 |
|  | Servings of diet soft drinks per day | 2505 | 365 | 0.870 | 0.726 | 1.044 | 0.1343 |
|  | Servings of regular (nondiet) soft drinks per day | 2507 | 365 | 0.926 | 0.686 | 1.249 | 0.6145 |
|  | Cups of coffee | 2508 | 365 | 0.967 | 0.925 | 1.010 | 0.1334 |
|  | Is coffee decaffeinated? | 1934 | 286 | 1.011 | 0.929 | 1.101 | 0.8012 |
| Physical activity | Time spent sitting for your job | 2487 | 358 | 0.998 | 0.873 | 1.141 | 0.9763 |
|  | Time spent standing for your job | 2480 | 360 | 0.926 | 0.806 | 1.065 | 0.2831 |
|  | Time spent walking for your job | 2479 | 362 | 1.034 | 0.895 | 1.195 | 0.6514 |
|  | Time spent doing light manual labor for your job | 2472 | 361 | 0.985 | 0.886 | 1.095 | 0.7827 |
|  | Time spent doing heavy manual labor for your job | 2462 | 355 | 1.039 | 0.922 | 1.171 | 0.5272 |
|  | Frequency of strenuous exercise in 7 day period | 2484 | 356 | 1.017 | 0.950 | 1.089 | 0.6227 |
|  | Frequency of moderate exercise in 7 day period | 2476 | 357 | 1.013 | 0.963 | 1.064 | 0.6222 |
|  | Frequency of mild exercise in 7 day period | 2469 | 357 | 1.027 | 0.981 | 1.076 | 0.2592 |
| Medical history | Any Allergies | 2482 | 353 | 1.012 | 0.801 | 1.278 | 0.9215 |
|  | Any food allergies | 2482 | 353 | 1.165 | 0.761 | 1.782 | 0.4820 |
|  | Any grass/pollen/dust allergies | 2482 | 353 | 0.929 | 0.715 | 1.205 | 0.5771 |
|  | Any allergies to pets | 2482 | 353 | 1.051 | 0.714 | 1.545 | 0.8020 |
|  | Any allergies to insect stings/bites | 2482 | 353 | 1.172 | 0.774 | 1.774 | 0.4533 |
|  | Any other allergies | 2482 | 353 | 1.123 | 0.866 | 1.455 | 0.3813 |
|  | Ever diagnosed with arthritis (osteoarthritis) | 2456 | 359 | 0.892 | 0.701 | 1.134 | 0.3502 |
|  | Ever diagnosed with arthritis (rheumatoid) | 2374 | 333 | 0.713 | 0.465 | 1.093 | 0.1203 |
|  | Ever diagnosed with fibromyalgia | 2452 | 350 | 0.471 | 0.225 | 0.984 | 0.0451 |
|  | Ever diagnosed with autoimmune disorder | 2446 | 352 | 1.416 | 0.781 | 2.567 | 0.2519 |
|  | Ever diagnosed with endometriosis | 2447 | 343 | 1.501 | 0.974 | 2.313 | 0.0654 |
|  | Ever diagnosed with hepatitis A, B, or C | 2476 | 357 | 1.052 | 0.509 | 2.174 | 0.8902 |
|  | Ever diagnosed with other liver disease | 2469 | 355 | 0.537 | 0.191 | 1.511 | 0.2389 |
|  | Ever had organ or bone marrow transplant | 2488 | 358 | . | . | . | 0.9733 |
|  | Ever diagnosed with bleeding disorder | 2482 | 357 | 1.460 | 0.711 | 2.998 | 0.3020 |
|  | Ever diagnosed with sickle cell anemia | 2472 | 357 | 1.321 | 0.149 | 11.741 | 0.8027 |
|  | Ever diagnosed with tuberculosis | 2482 | 356 | 0.667 | 0.152 | 2.920 | 0.5911 |
|  | Ever diagnosed with thyroid cancer | 2490 | 357 | 1.400 | 0.464 | 4.223 | 0.5499 |
|  | Ever diagnosed with lung cancer | 2483 | 355 | 1.240 | 0.493 | 3.119 | 0.6483 |
|  | Ever diagnosed with breast cancer | 1472 | 166 | 1.449 | 0.926 | 2.268 | 0.1048 |
|  | Ever diagnosed with esophageal cancer | 2475 | 357 | . | . | . | 0.9737 |
|  | Ever diagnosed with pancreatic cancer | 2490 | 356 | 1.161 | 0.138 | 9.760 | 0.8906 |
|  | Ever diagnosed with stomach cancer | 2482 | 356 | 2.667 | 0.427 | 16.658 | 0.2940 |
|  | Ever diagnosed with colon/rectal cancer | 2489 | 358 | 0.700 | 0.290 | 1.689 | 0.4274 |
|  | Ever diagnosed with liver cancer | 2482 | 354 | 0.738 | 0.088 | 6.156 | 0.7786 |
|  | Ever diagnosed with uterine/endometrial cancer | 1470 | 164 | 1.251 | 0.425 | 3.689 | 0.6842 |
|  | Ever diagnosed with cervical cancer | 1471 | 165 | 0.958 | 0.331 | 2.770 | 0.9367 |
|  | Ever diagnosed with ovarian cancer | 1470 | 165 | 1.703 | 0.370 | 7.829 | 0.4941 |
|  | Ever diagnosed with prostate cancer | 1013 | 190 | 0.901 | 0.565 | 1.439 | 0.6638 |
|  | Ever diagnosed with testicular cancer | 1017 | 194 | 1.335 | 0.278 | 6.414 | 0.7185 |
|  | Ever diagnosed with melanoma | 2469 | 357 | 1.275 | 0.766 | 2.120 | 0.3501 |
|  | Ever diagnosed with non-melanoma skin cancer | 2476 | 357 | 1.024 | 0.758 | 1.382 | 0.8794 |
|  | Ever diagnosed with sarcoma | 2469 | 352 | 1.563 | 0.717 | 3.408 | 0.2618 |
|  | Ever diagnosed with bone cancer | 2494 | 358 | 2.640 | 0.438 | 15.909 | 0.2895 |
|  | Ever diagnosed with kidney cancer | 2487 | 358 | 2.566 | 0.782 | 8.418 | 0.1201 |
|  | Ever diagnosed with urinary/bladder cancer | 2483 | 357 | 1.167 | 0.447 | 3.045 | 0.7519 |
|  | Ever diagnosed with other cancer | 2459 | 356 | 0.977 | 0.426 | 2.243 | 0.9569 |
|  | Ever diagnosed with Alzheimer's disease | 2488 | 358 | . | . | . | 0.9808 |
|  | Ever diagnosed with Parkinson's disease | 2486 | 358 | 0.644 | 0.144 | 2.882 | 0.5650 |
|  | Ever diagnosed with dementia | 2481 | 356 | 0.940 | 0.260 | 3.404 | 0.9249 |
|  | Ever diagnosed with migraine headaches | 2484 | 355 | 1.031 | 0.747 | 1.424 | 0.8506 |
|  | Ever diagnosed with stroke (CVA) | 2481 | 357 | 1.014 | 0.462 | 2.225 | 0.9730 |
|  | Ever diagnosed with TIA | 2474 | 354 | 0.968 | 0.533 | 1.758 | 0.9148 |
|  | Ever diagnosed with epilepsy | 2490 | 355 | 0.315 | 0.075 | 1.323 | 0.1147 |
|  | Ever diagnosed with narcolepsy | 2490 | 355 | 0.924 | 0.208 | 4.100 | 0.9175 |
|  | Ever diagnosed with anxiety | 2486 | 356 | 1.016 | 0.736 | 1.403 | 0.9228 |
|  | Ever diagnosed with depression | 2476 | 353 | 0.899 | 0.663 | 1.219 | 0.4929 |
|  | Ever diagnosed with Down syndrome | 2490 | 356 | . | . | . | 0.9782 |
|  | Ever diagnosed with bipolar disorder | 2485 | 356 | 1.197 | 0.263 | 5.447 | 0.8162 |
|  | Ever diagnosed with ADHD | 2483 | 355 | 1.360 | 0.630 | 2.937 | 0.4334 |
|  | Ever diagnosed with other psychiatric/metal illness | 2479 | 355 | 1.291 | 0.496 | 3.358 | 0.6004 |
|  | Ever diagnosed with glaucoma | 2481 | 353 | 0.851 | 0.520 | 1.393 | 0.5209 |
|  | Ever diagnosed with cataracts | 2484 | 355 | 0.989 | 0.758 | 1.290 | 0.9335 |
|  | Ever diagnosed with abnormal distance vision | 2451 | 353 | 0.788 | 0.587 | 1.059 | 0.1143 |
|  | Ever diagnosed with lazy eye (amblyopia) | 2455 | 357 | 1.100 | 0.622 | 1.945 | 0.7442 |
|  | Ever diagnosed with crossing/wandering eyes | 2483 | 353 | 1.008 | 0.490 | 2.073 | 0.9829 |
|  | Ever diagnosed with macular degeneration | 2470 | 354 | 0.523 | 0.278 | 0.986 | 0.0452 |
|  | Ever diagnosed with heart attack/MI | 2478 | 354 | 0.938 | 0.602 | 1.461 | 0.7762 |
|  | Ever diagnosed with CHF | 2465 | 353 | 0.593 | 0.276 | 1.274 | 0.1805 |
|  | Ever diagnosed with atrial fibrillation/arrhthmia | 2467 | 350 | 1.027 | 0.708 | 1.489 | 0.8890 |
|  | Ever diagnosed with congenital heart disease | 2449 | 352 | 0.881 | 0.389 | 1.995 | 0.7613 |
|  | Ever diagnosed with high blood pressure | 2486 | 360 | 0.925 | 0.732 | 1.167 | 0.5092 |
|  | Ever diagnosed with high cholesterol | 2479 | 355 | 1.062 | 0.844 | 1.336 | 0.6082 |
|  | Ever diagnosed with venous thromboembolism | 1106 | 158 | 0.842 | 0.365 | 1.943 | 0.6867 |
|  | Ever diagnosed with coronary artery disease | 1356 | 193 | 1.229 | 0.758 | 1.993 | 0.4041 |
|  | Ever diagnosed with blood clots in a vein | 1351 | 191 | 0.780 | 0.432 | 1.412 | 0.4123 |
|  | Ever diagnosed with asthma | 2481 | 355 | 0.785 | 0.530 | 1.162 | 0.2265 |
|  | Ever diagnosed with COPD | 2473 | 356 | 1.116 | 0.667 | 1.868 | 0.6753 |
|  | Ever diagnosed with sleep apnea | 2482 | 354 | 0.927 | 0.689 | 1.248 | 0.6185 |
|  | Ever diagnosed with asbestosis | 2462 | 350 | 0.565 | 0.125 | 2.545 | 0.4571 |
|  | Ever diagnosed with pulmonary fibrosis | 2459 | 348 | 1.590 | 0.502 | 5.038 | 0.4307 |
|  | Ever diagnosed with acid reflux / GERD | 2480 | 356 | 1.121 | 0.873 | 1.439 | 0.3723 |
|  | Ever diagnosed with Barrett's esophagus | 2462 | 355 | 1.457 | 0.720 | 2.950 | 0.2957 |
|  | Ever diagnosed with celiac disease | 2479 | 354 | 0.524 | 0.122 | 2.252 | 0.3851 |
|  | Ever diagnosed with irritable bowel syndrome | 2471 | 355 | 0.995 | 0.663 | 1.494 | 0.9823 |
|  | Ever diagnosed with Crohn's disease | 2487 | 355 | 0.800 | 0.336 | 1.906 | 0.6152 |
|  | Ever diagnosed with Lynch syndrome / HNPCC | 2476 | 353 | 3.546 | 0.675 | 18.637 | 0.1349 |
|  | Ever diagnosed with type 1 diabetes | 2438 | 350 | 0.471 | 0.108 | 2.051 | 0.3161 |
|  | Ever diagnosed with type 2 diabetes | 2471 | 358 | 0.785 | 0.544 | 1.135 | 0.1980 |
|  | Ever diagnosed with hyperthyroidism/hypothyroidism | 2429 | 351 | 1.166 | 0.829 | 1.641 | 0.3772 |
|  | Ever diagnosed with a cancer other than lymphoma/leukemia | 2502 | 363 | 1.222 | 0.964 | 1.549 | 0.0982 |
| Family history | Any 1st degree relative with leukemia/lymphoma | 2169 | 305 | 1.435 | 0.988 | 2.085 | 0.0579 |

*Adjusted for age and sex

**Supplemental Table 2**: List of 41 CLL susceptibility SNPs and the association with MBL and MBL subtypes among European Ancestry Individuals

| Locus | Nearest Gene(s) | Lead SNP in | Position | Risk | Results from a  published study  (effect sizes used for  the PRS calculations)^1,2^ | | MBL overall  N_CLL_=560/N_control_=2631 | | | | LC-MBL  N_CLL_=396/N_control_=2631 | | | | HC-MBL  N_CLL_=164/N_control_=2631 | | | |
| --- | --- | --- | --- | --- | --- | --- | --- | --- | --- | --- | --- | --- | --- | --- | --- | --- | --- | --- |
|  |  | Published fine  mapping GWAS | (hg19, bp) | Allele | OR (95% CI) | *P* | OR^§^ | 95% CI | | *P* | OR^§^ | 95% CI | | *P* | OR^§^ | 95% CI | | *P* |
| 1p36.11 | MDS2 | rs34676223 | 23943735 | C | 1.19 (1.14-1.25) | 5.04E-13 | 1.09 | 0.93 | 1.27 | 0.29 | 1.15 | 0.96 | 1.37 | 0.13 | 0.96 | 0.72 | 1.26 | 0.75 |
| 1q42.13 | RHOU | rs41271473 | 228880296 | G | 1.19 (1.13-1.26) | 1.06E-10 | 0.97 | 0.82 | 1.15 | 0.72 | 1.05 | 0.86 | 1.27 | 0.65 | 0.91 | 0.66 | 1.24 | 0.53 |
| 2p22.2 | *QPCT, PRKD3* | rs888096 | 37,603,801 | A | 1.15 (1.09-1.21) | 5.20E-08 | 1.07 | 0.93 | 1.23 | 0.34 | 1.11 | 0.95 | 1.29 | 0.21 | 0.96 | 0.74 | 1.23 | 0.72 |
| 2q13 | *ACOXL, BCL2L11* | rs1002015 | 111,616,619 | C | 1.30 (1.23-1.37) | 2.23E-23 | **1.26** | **1.10** | **1.45** | **8.9x10^-4^** | 1.22 | **1.04** | **1.42** | **0.01** | **1.48** | **1.15** | **1.92** | **0.003** |
|  |  | rs58055674 | 111,831,793 | C | 1.41 (1.32-1.5) | 2.02E-27 | **1.26** | **1.06** | **1.50** | **0.01** | 1.10 | 0.90 | 1.34 | 0.37 | **1.77** | **1.32** | **2.37** | **1.6x10^-4^** |
|  |  | rs6708784 | 111,927,379 | G | 1.30 (1.24-1.37) | 2.67E-25 | **1.15** | **1.00** | **1.31** | **0.05** | 1.14 | 0.97 | 1.33 | 0.10 | 1.17 | 0.91 | 1.51 | 0.21 |
| 2q33.1 | *CASP10/CASP8* | rs7558911 | 202,023,949 | A | 1.18 (1.12-1.24) | 5.05E-11 | **1.21** | **1.05** | **1.38** | **0.01** | 1.18 | **1.01** | **1.38** | **0.04** | 1.23 | 0.96 | 1.59 | 0.11 |
| 2q37.1 | *SP110, SP140* | rs34004493 | 231,154,012 | G | 1.39 (1.31-1.47) | 3.67E-32 | **1.45** | **1.25** | **1.68** | **1.1x10^-6^** | 1.43 | **1.21** | **1.69** | **2.9x10^-5^** | **1.50** | **1.15** | **1.95** | **0.003** |
| 2q37.3 | *FARP2* | rs757978 | 242,371,101 | T | 1.29 (1.2-1.39) | 5.80E-11 | 1.17 | 0.96 | 1.44 | 0.12 | 1.16 | 0.91 | 1.46 | 0.23 | 1.32 | 0.93 | 1.88 | 0.12 |
| 3p24.1 | *EOMES* | rs9880772 | 27,777,779 | A | 1.16 (1.11-1.22) | 1.91E-09 | 1.00 | 0.87 | 1.15 | 0.98 | 0.96 | 0.82 | 1.12 | 0.63 | 1.04 | 0.81 | 1.35 | 0.75 |
| 3q26.2 | *MYNN, TERC* | rs1317082 | 169,497,585 | A | 1.19 (1.12-1.26) | 5.77E-09 | **1.24** | **1.05** | **1.46** | **0.01** | 1.19 | 0.99 | 1.43 | 0.07 | 1.36 | 0.99 | 1.87 | 0.05 |
| 4q24 | BANK1 | rs13107612* | 102739980 | C | 1.17 (1.11-1.22) | 1.37E-10 | 1.08 | 0.93 | 1.26 | 0.28 | 1.12 | 0.94 | 1.32 | 0.20 | 1.04 | 0.79 | 1.37 | 0.78 |
| 4q25 | *LEF1* | rs7690934 | 109,025,865 | C | 1.16 (1.11-1.22) | 6.08E-09 | 1.07 | 0.93 | 1.23 | 0.33 | 1.01 | 0.86 | 1.18 | 0.89 | 1.19 | 0.92 | 1.54 | 0.19 |
| 4q35.1 | LOC728175 | rs57214277 | 185254772 | T | 1.13 (1.08-1.18) | 3.69E-08 | **1.17** | **1.02** | **1.34** | **0.02** | 1.15 | 0.99 | 1.35 | 0.07 | 1.18 | 0.92 | 1.52 | 0.18 |
| 5p15.33 | *TERT* | rs7705526 | 1,285,974 | A | 1.18 (1.12-1.25) | 5.90E-10 | **1.17** | **1.02** | **1.35** | **0.03** | 1.27 | **1.09** | **1.49** | **0.003** | 0.93 | 0.71 | 1.21 | 0.59 |
| 6p25.3 | *IRF4* | rs9392504 | 412,802 | A | 1.33 (1.26-1.4) | 9.81E-29 | **1.26** | **1.10** | **1.44** | **0.001** | 1.19 | **1.02** | **1.39** | **0.03** | **1.41** | **1.10** | **1.82** | **0.01** |
| 6p21.32 | *HLA* | rs9271176 | 32,578,127 | G | 1.29 (1.22-1.36) | 3.16E-20 | 1.12 | 0.96 | 1.30 | 0.14 | 1.11 | 0.94 | 1.31 | 0.24 | 1.09 | 0.83 | 1.43 | 0.52 |
| 6p21.31 | *BAK1* | rs210143 | 33,546,930 | C | 1.26 (1.19-1.33) | 5.77E-16 | 1.03 | 0.89 | 1.20 | 0.69 | 1.01 | 0.85 | 1.20 | 0.88 | 1.10 | 0.83 | 1.45 | 0.52 |
| 6p21.31 | C6orf106 | rs3800461 | 34616322 | C | 1.20 (1.13-1.28) | 1.97E-08 | 1.11 | 0.90 | 1.37 | 0.31 | 1.07 | 0.85 | 1.35 | 0.58 | 1.06 | 0.73 | 1.55 | 0.75 |
| 6q25.2 | *IPCEF1* | rs4869818 | 154,471,225 | G | 1.15 (1.09-1.21) | 4.11E-08 | 1.00 | 0.88 | 1.15 | 0.97 | 1.00 | 0.86 | 1.16 | 0.98 | 0.98 | 0.76 | 1.25 | 0.85 |
| 7q31.33 | *POT1* | rs2267708 | 124,392,512 | T | 1.16 (1.1-1.22) | 8.55E-09 | **1.26** | **1.10** | **1.44** | **0.001** | 1.22 | **1.05** | **1.42** | **0.01** | **1.32** | **1.02** | **1.69** | **0.03** |
| 8q22.3 | *ODF1* | rs2511713 | 103,577,865 | G | 1.17 (1.1-1.23) | 6.04E-08 | **1.30** | **1.12** | **1.51** | **0.001** | 1.22 | **1.03** | **1.45** | **0.02** | **1.56** | **1.20** | **2.04** | **0.001** |
| 8q24.21 | *POU5F1B* | rs2466029 | 128,200,971 | G | 1.23 (1.17-1.3) | 7.47E-16 | **1.15** | **1.00** | **1.31** | **0.05** | 1.27 | **1.09** | **1.48** | **0.003** | 0.89 | 0.69 | 1.15 | 0.38 |
| 9p21.3 | *CDKN2B-AS1* | rs1679013 | 22,206,987 | C | 1.16 (1.1-1.22) | 2.17E-08 | **1.16** | **1.02** | **1.33** | **0.03** | 1.10 | 0.94 | 1.29 | 0.22 | 1.28 | 1.00 | 1.65 | 0.05 |
| 10q23.31 | *ACTA, FAS* | rs6586163 | 90,752,018 | A | 1.23 (1.17-1.29) | 1.14E-15 | **1.22** | **1.06** | **1.40** | **0.004** | 1.35 | **1.16** | **1.58** | **1.4x10^-4^** | 0.92 | 0.72 | 1.18 | 0.51 |
| 11p15.5 | *C11orf21, TSPAN32* | rs2651823 | 2,321,650 | A | 1.18 (1.13-1.25) | 5.24E-11 | 1.02 | 0.89 | 1.17 | 0.81 | 1.01 | 0.87 | 1.18 | 0.90 | 1.01 | 0.79 | 1.29 | 0.95 |
| 11q23.2 | TMPRSS5 | rs61904987 | 113517203 | T | 1.24 (1.16-1.32) | 2.46E-11 | 1.08 | 0.88 | 1.32 | 0.48 | 1.02 | 0.81 | 1.29 | 0.88 | 1.15 | 0.80 | 1.65 | 0.45 |
| 11q24.1 | *SCN3B, GRAMD1B* | rs35923643 | 123,355,391 | G | 1.63 (1.53-1.72) | 4.26E-58 | **1.48** | **1.26** | **1.73** | **9.1x10^-7^** | 1.42 | **1.19** | **1.69** | **1.2x10^-4^** | **1.53** | **1.16** | **2.01** | **0.003** |
| 12q24.13 | *OAS3* | rs6489882 | 113,381,376 | G | 1.16 (1.1-1.22) | 4.76E-08 | 1.14 | 0.99 | 1.31 | 0.07 | 1.15 | 0.98 | 1.35 | 0.08 | 1.07 | 0.82 | 1.39 | 0.62 |
| 15q15.1 | *BMF, BUB1B* | rs8024033 | 40,403,657 | C | 1.26 (1.2-1.32) | 7.13E-19 | 1.10 | 0.96 | 1.26 | 0.16 | 1.07 | 0.92 | 1.25 | 0.37 | 1.15 | 0.89 | 1.47 | 0.28 |
| 15q21.3 | *RFX7, NEDD4* | rs142215530 | 56,777,691 | G | 1.39 (1.29-1.5) | 2.46E-18 | 1.20 | 0.97 | 1.48 | 0.09 | 1.15 | 0.91 | 1.46 | 0.23 | 1.31 | 0.90 | 1.92 | 0.16 |
| 15q23 | *RPLP1* | rs11637565 | 70,020,525 | G | 1.35 (1.28-1.42) | 1.96E-31 | **1.22** | **1.06** | **1.41** | **0.01** | 1.17 | 0.99 | 1.37 | 0.06 | **1.32** | **1.02** | **1.71** | **0.03** |
| 16q24.1 | *IRF8* | rs391855 | 85,928,621 | A | 1.34 (1.27-1.41) | 1.25E-28 | **1.36** | **1.18** | **1.56** | **1.9x10^-5^** | 1.30 | **1.11** | **1.53** | **0.001** | **1.44** | **1.11** | **1.87** | **0.01** |
|  |  | rs305065 | 85,973,866 | C | 1.16 (1.1-1.22) | 7.57E-08 | 0.95 | 0.82 | 1.10 | 0.47 | 0.95 | 0.81 | 1.12 | 0.58 | 0.90 | 0.69 | 1.17 | 0.44 |
| 18q21.1 | CXXC1 | rs1036935 | 47843534 | A | 1.15 (1.10-1.21) | 3.27E-08 | 1.02 | 0.87 | 1.20 | 0.79 | 1.01 | 0.84 | 1.21 | 0.95 | 1.10 | 0.83 | 1.47 | 0.50 |
| 18q21.32 | *PMAIP1* | rs4368253 | 57,622,287 | C | 1.17 (1.11-1.24) | 1.26E-08 | **1.17** | **1.01** | **1.35** | **0.04** | 1.16 | 0.98 | 1.37 | 0.08 | 1.16 | 0.88 | 1.51 | 0.30 |
| 18q21.33 | *BCL2* | rs77551289 | 60,788,745 | A | 1.37 (1.25-1.5) | 1.83E-11 | **1.32** | **1.03** | **1.69** | **0.03** | 1.26 | 0.95 | 1.67 | 0.11 | 1.50 | 0.93 | 2.41 | 0.10 |
|  |  | rs4987852 | 60,793,921 | C | 1.32 (1.2-1.44) | 4.66E-09 | **1.32** | **1.03** | **1.69** | **0.03** | 1.41 | **1.07** | **1.87** | **0.02** | 1.13 | 0.71 | 1.81 | 0.61 |
| 19p13.3 | ZBTB7A | rs7254272 | 4069119 | A | 1.17 (1.10-1.73) | 4.67E-08 | 1.08 | 0.90 | 1.29 | 0.43 | 1.02 | 0.83 | 1.26 | 0.84 | 1.24 | 0.90 | 1.71 | 0.19 |
| 19q13.3 | *PRKD2, STRN4* | rs874460 | 47,176,752 | C | 1.24 (1.15-1.34) | 3.37E-08 | 1.12 | 0.92 | 1.36 | 0.27 | 1.13 | 0.90 | 1.41 | 0.30 | 0.98 | 0.69 | 1.38 | 0.90 |
| 22q13.33 | NCAPH2 | rs140522 | 50971266 | T | 1.15 (1.10-1.20) | 2.70E-09 | **1.21** | **1.05** | **1.40** | **0.01** | 1.05 | 0.89 | 1.24 | 0.57 | **1.75** | **1.35** | **2.26** | **2.4x10^-5^** |

CLL=chronic lymphocytic leukemia; MBL=monoclonal B-cell lymphocytosis; OR=Odds Ratio; CI=Confidence Interval; LD=Linkage Disequilibrium; ^§^Adjusted for age, sex and PC3; *Proxy for rs71597109; **Bold,** statistically significant P<0.05

**Supplemental Table 3**: List of 41 CLL susceptibility SNPs and the association with CLL risk among European Ancestry and African American Individuals

| Locus | Nearest Gene(s) | Lead SNP in | Position | Risk | Results from a  published study  (effect sizes used for  the PRS calculations)^1,2^ | | EA CLL  N_CLL_=696/N_control_=2631 | | | | African American CLL  N_CLL_=173/N_control_=235 | | | |
| --- | --- | --- | --- | --- | --- | --- | --- | --- | --- | --- | --- | --- | --- | --- |
|  |  | Published fine  mapping GWAS | (hg19, bp) | Allele | OR (95% CI) | *P* | OR^§^ | 95% CI | | *P* | OR^§^ | 95% CI | | *P* |
| 1p36.11 | MDS2 | rs34676223 | 23943735 | C | 1.19 (1.14-1.25) | 5.04E-13 | 1.10 | 0.94 | 1.28 | 0.24 | 0.98 | 0.70 | 1.37 | 0.92 |
| 1q42.13 | RHOU | rs41271473 | 228880296 | G | 1.19 (1.13-1.26) | 1.06E-10 | 0.94 | 0.79 | 1.11 | 0.45 | 1.16 | 0.75 | 1.78 | 0.51 |
| 2p22.2 | *QPCT, PRKD3* | rs888096 | 37,603,801 | A | 1.15 (1.09-1.21) | 5.20E-08 | 1.19 | 1.04 | 1.37 | **0.014** | 1.38 | 0.88 | 2.16 | 0.16 |
| 2q13 | *ACOXL, BCL2L11* | rs1002015 | 111,616,619 | C | 1.30 (1.23-1.37) | 2.23E-23 | 1.39 | 1.21 | 1.61 | **5.1E-06** | 0.95 | 0.66 | 1.36 | 0.78 |
|  |  | rs58055674 | 111,831,793 | C | 1.41 (1.32-1.5) | 2.02E-27 | 1.69 | 1.42 | 2.01 | **2.8E-09** | 1.66 | 0.92 | 3.02 | 0.09 |
|  |  | rs6708784 | 111,927,379 | G | 1.30 (1.24-1.37) | 2.67E-25 | 1.27 | 1.10 | 1.46 | **0.001** | 1.03 | 0.74 | 1.45 | 0.86 |
| 2q33.1 | *CASP10/CASP8* | rs7558911 | 202,023,949 | A | 1.18 (1.12-1.24) | 5.05E-11 | 1.06 | 0.92 | 1.21 | 0.44 | 1.10 | 0.78 | 1.55 | 0.58 |
| 2q37.1 | *SP110, SP140* | rs34004493 | 231,154,012 | G | 1.39 (1.31-1.47) | 3.67E-32 | 1.47 | 1.26 | 1.71 | **5.6E-07** | 1.06 | 0.64 | 1.73 | 0.83 |
| 2q37.3 | *FARP2* | rs757978 | 242,371,101 | T | 1.29 (1.2-1.39) | 5.80E-11 | 1.25 | 1.01 | 1.53 | **0.038** | 1.09 | 0.64 | 1.85 | 0.75 |
| 3p24.1 | *EOMES* | rs9880772 | 27,777,779 | A | 1.16 (1.11-1.22) | 1.91E-09 | 1.16 | 1.01 | 1.33 | **0.034** | 0.80 | 0.55 | 1.17 | 0.26 |
| 3q26.2 | *MYNN, TERC* | rs1317082 | 169,497,585 | A | 1.19 (1.12-1.26) | 5.77E-09 | 1.33 | 1.12 | 1.58 | **0.001** | 1.16 | 0.56 | 2.38 | 0.69 |
| 4q24 | BANK1 | rs13107612* | 102739980 | C | 1.17 (1.11-1.22) | 1.37E-10 | 1.05 | 0.91 | 1.22 | 0.502 | 1.30 | 0.90 | 1.87 | 0.16 |
| 4q25 | *LEF1* | rs7690934 | 109,025,865 | C | 1.16 (1.11-1.22) | 6.08E-09 | 1.27 | 1.10 | 1.46 | **0.001** | **1.41** | **1.03** | **1.95** | **0.03** |
| 4q35.1 | LOC728175 | rs57214277 | 185254772 | T | 1.13 (1.08-1.18) | 3.69E-08 | 1.21 | 1.05 | 1.38 | **0.008** | 1.20 | 0.72 | 1.99 | 0.48 |
| 5p15.33 | *TERT* | rs7705526 | 1,285,974 | A | 1.18 (1.12-1.25) | 5.90E-10 | 1.29 | 1.12 | 1.49 | **4.9E-04** | 0.83 | 0.56 | 1.23 | 0.35 |
| 6p25.3 | *IRF4* | rs9392504 | 412,802 | A | 1.33 (1.26-1.4) | 9.81E-29 | 1.46 | 1.27 | 1.68 | **1.6E-07** | 0.90 | 0.62 | 1.31 | 0.58 |
| 6p21.32 | *HLA* | rs9271176 | 32,578,127 | G | 1.29 (1.22-1.36) | 3.16E-20 | 1.28 | 1.10 | 1.50 | **0.001** | 1.08 | 0.74 | 1.59 | 0.67 |
| 6p21.31 | *BAK1* | rs210143 | 33,546,930 | C | 1.26 (1.19-1.33) | 5.77E-16 | 1.22 | 1.04 | 1.42 | **0.013** | 0.85 | 0.59 | 1.22 | 0.38 |
| 6p21.31 | C6orf106 | rs3800461 | 34616322 | C | 1.20 (1.13-1.28) | 1.97E-08 | 1.09 | 0.89 | 1.33 | 0.42 | 1.44 | 0.67 | 3.08 | 0.35 |
| 6q25.2 | *IPCEF1* | rs4869818 | 154,471,225 | G | 1.15 (1.09-1.21) | 4.11E-08 | 1.24 | 1.08 | 1.42 | **0.002** | 0.94 | 0.67 | 1.31 | 0.69 |
| 7q31.33 | *POT1* | rs2267708 | 124,392,512 | T | 1.16 (1.1-1.22) | 8.55E-09 | 1.20 | 1.04 | 1.37 | **0.010** | 1.03 | 0.75 | 1.42 | 0.85 |
| 8q22.3 | *ODF1* | rs2511713 | 103,577,865 | G | 1.17 (1.1-1.23) | 6.04E-08 | 1.23 | 1.05 | 1.43 | **0.009** | 1.14 | 0.81 | 1.62 | 0.45 |
| 8q24.21 | *POU5F1B* | rs2466029 | 128,200,971 | G | 1.23 (1.17-1.3) | 7.47E-16 | 1.08 | 0.94 | 1.24 | 0.26 | 0.72 | 0.48 | 1.06 | 0.10 |
| 9p21.3 | *CDKN2B-AS1* | rs1679013 | 22,206,987 | C | 1.16 (1.1-1.22) | 2.17E-08 | 1.13 | 0.98 | 1.29 | 0.10 | **1.56** | **1.08** | **2.25** | **0.02** |
| 10q23.31 | *ACTA, FAS* | rs6586163 | 90,752,018 | A | 1.23 (1.17-1.29) | 1.14E-15 | 1.17 | 1.02 | 1.34 | **0.027** | 0.85 | 0.60 | 1.22 | 0.39 |
| 11p15.5 | *C11orf21, TSPAN32* | rs2651823 | 2,321,650 | A | 1.18 (1.13-1.25) | 5.24E-11 | 1.17 | 1.02 | 1.34 | **0.026** | 1.30 | 0.84 | 2.01 | 0.24 |
| 11q23.2 | TMPRSS5 | rs61904987 | 113517203 | T | 1.24 (1.16-1.32) | 2.46E-11 | 1.26 | 1.03 | 1.53 | **0.022** | 2.11 | 0.61 | 7.24 | 0.24 |
| 11q24.1 | *SCN3B, GRAMD1B* | rs35923643 | 123,355,391 | G | 1.63 (1.53-1.72) | 4.26E-58 | 1.83 | 1.56 | 2.14 | **5.2E-14** | 1.53 | 0.77 | 3.04 | 0.23 |
| 12q24.13 | *OAS3* | rs6489882 | 113,381,376 | G | 1.16 (1.1-1.22) | 4.76E-08 | 1.15 | 1.00 | 1.32 | 0.06 | 0.99 | 0.52 | 1.87 | 0.98 |
| 15q15.1 | *BMF, BUB1B* | rs8024033 | 40,403,657 | C | 1.26 (1.2-1.32) | 7.13E-19 | 1.24 | 1.09 | 1.43 | **0.002** | 0.97 | 0.70 | 1.33 | 0.85 |
| 15q21.3 | *RFX7, NEDD4* | rs142215530 | 56,777,691 | G | 1.39 (1.29-1.5) | 2.46E-18 | 1.37 | 1.11 | 1.69 | **0.003** | 0.46 | 0.17 | 1.26 | 0.13 |
| 15q23 | *RPLP1* | rs11637565 | 70,020,525 | G | 1.35 (1.28-1.42) | 1.96E-31 | 1.34 | 1.16 | 1.55 | **6.6E-05** | 0.80 | 0.56 | 1.14 | 0.22 |
| 16q24.1 | *IRF8* | rs391855 | 85,928,621 | A | 1.34 (1.27-1.41) | 1.25E-28 | 1.17 | 1.02 | 1.35 | **0.026** | 1.01 | 0.71 | 1.43 | 0.95 |
|  |  | rs305065 | 85,973,866 | C | 1.16 (1.1-1.22) | 7.57E-08 | 1.22 | 1.05 | 1.41 | **0.008** | 1.12 | 0.61 | 2.03 | 0.72 |
| 18q21.1 | CXXC1 | rs1036935 | 47843534 | A | 1.15 (1.10-1.21) | 3.27E-08 | 1.39 | 1.19 | 1.63 | **3.5E-05** | 0.84 | 0.59 | 1.20 | 0.33 |
| 18q21.32 | *PMAIP1* | rs4368253 | 57,622,287 | C | 1.17 (1.11-1.24) | 1.26E-08 | 1.33 | 1.15 | 1.55 | **2.1E-04** | 0.71 | 0.49 | 1.01 | 0.06 |
| 18q21.33 | *BCL2* | rs77551289 | 60,788,745 | A | 1.37 (1.25-1.5) | 1.83E-11 | 1.60 | 1.23 | 2.08 | **4.2E-04** | 0.70 | 0.19 | 2.62 | 0.60 |
|  |  | rs4987852 | 60,793,921 | C | 1.32 (1.2-1.44) | 4.66E-09 | 1.60 | 1.25 | 2.04 | **1.6E-04** | 0.55 | 0.09 | 3.41 | 0.52 |
| 19p13.3 | ZBTB7A | rs7254272 | 4069119 | A | 1.17 (1.10-1.73) | 4.67E-08 | 1.10 | 0.91 | 1.32 | 0.33 | 0.83 | 0.60 | 1.17 | 0.29 |
| 19q13.3 | *PRKD2, STRN4* | rs874460 | 47,176,752 | C | 1.24 (1.15-1.34) | 3.37E-08 | 1.36 | 1.11 | 1.68 | **0.003** | 1.60 | 0.80 | 3.17 | 0.18 |
| 22q13.33 | NCAPH2 | rs140522 | 50971266 | T | 1.15 (1.10-1.20) | 2.70E-09 | 1.17 | 1.01 | 1.35 | **0.034** | 0.85 | 0.62 | 1.17 | 0.33 |

CLL=chronic lymphocytic leukemia; EA=European Ancestry; AA=African American; OR=Odds Ratio; CI=Confidence Interval; LD=Linkage Disequilibrium; ^§^Adjusted for age, sex and PC3; *Proxy for rs71597109; **Bold,** statistically significant P<0.05

**Supplemental Table 4:** Minor Allele frequency of 41 CLL susceptibility SNPs by ethnicity in the 1000G and current study

| CHR | SNP | REF | ALT | 1000G ALTF.EA | 1000G  ALTF.AA | AAF.ctrl.AA | AAF.ctrl.EA | AAF.case.AA | AAF.case.EA |
| --- | --- | --- | --- | --- | --- | --- | --- | --- | --- |
| 1 | rs34676223 | C | A | 0.32 | 0.41 | 0.37 | 0.29 | 0.38 | 0.28 |
| 1 | rs41271473 | G | A | 0.19 | 0.22 | 0.17 | 0.20 | 0.14 | 0.20 |
| 2 | rs888096 | G | A | 0.42 | 0.99 | 0.87 | 0.37 | 0.83 | 0.40 |
| 2 | rs1002015 | T | C | 0.53 | 0.29 | 0.30 | 0.56 | 0.29 | 0.63 |
| 2 | rs58055674 | T | C | 0.17 | 0.03 | 0.06 | 0.17 | 0.10 | 0.24 |
| 2 | rs6708784 | A | G | 0.51 | 0.32 | 0.41 | 0.51 | 0.41 | 0.56 |
| 2 | rs7558911 | A | G | 0.42 | 0.38 | 0.40 | 0.48 | 0.42 | 0.46 |
| 2 | rs34004493 | A | G | 0.27 | 0.07 | 0.11 | 0.26 | 0.13 | 0.34 |
| 2 | rs757978 | C | T | 0.14 | 0.10 | 0.11 | 0.10 | 0.10 | 0.13 |
| 3 | rs9880772 | G | A | 0.47 | 0.87 | 0.75 | 0.46 | 0.77 | 0.51 |
| 3 | rs1317082 | A | G | 0.26 | 0.01 | 0.06 | 0.25 | 0.05 | 0.20 |
| 4 | rs13107612 | C | T | 0.27 | 0.38 | 0.32 | 0.31 | 0.28 | 0.30 |
| 4 | rs7690934 | T | C | 0.63 | 0.56 | 0.59 | 0.58 | 0.54 | 0.64 |
| 4 | rs57214277 | C | T | 0.41 | 0.00 | 0.11 | 0.41 | 0.12 | 0.45 |
| 5 | rs7705526 | C | A | 0.32 | 0.14 | 0.20 | 0.33 | 0.19 | 0.39 |
| 6 | rs9392504 | G | A | 0.51 | 0.24 | 0.26 | 0.51 | 0.23 | 0.60 |
| 6 | rs9271176 | A | G | 0.67 | 0.70 | 0.75 | 0.67 | 0.74 | 0.70 |
| 6 | rs210143 | T | C | 0.70 | 0.78 | 0.72 | 0.70 | 0.73 | 0.75 |
| 6 | rs3800461 | G | C | 0.11 | 0.01 | 0.03 | 0.13 | 0.04 | 0.12 |
| 6 | rs4869818 | G | A | 0.59 | 0.56 | 0.55 | 0.56 | 0.55 | 0.53 |
| 7 | rs2267708 | C | T | 0.50 | 0.50 | 0.51 | 0.44 | 0.48 | 0.48 |
| 8 | rs2511713 | A | G | 0.25 | 0.24 | 0.27 | 0.26 | 0.31 | 0.30 |
| 8 | rs2466029 | C | G | 0.39 | 0.74 | 0.74 | 0.38 | 0.77 | 0.40 |
| 9 | rs1679013 | C | T | 0.46 | 0.29 | 0.33 | 0.48 | 0.25 | 0.46 |
| 10 | rs6586163 | A | C | 0.43 | 0.89 | 0.78 | 0.50 | 0.74 | 0.46 |
| 11 | rs2651823 | A | G | 0.58 | 0.91 | 0.79 | 0.58 | 0.81 | 0.53 |
| 11 | rs61904987 | C | T | 0.13 | 0.00 | 0.01 | 0.11 | 0.03 | 0.14 |
| 11 | rs35923643 | A | G | 0.17 | 0.02 | 0.05 | 0.20 | 0.07 | 0.31 |
| 12 | rs6489882 | G | A | 0.63 | 1.00 | 0.93 | 0.65 | 0.91 | 0.62 |
| 15 | rs8024033 | C | G | 0.50 | 0.66 | 0.56 | 0.50 | 0.56 | 0.44 |
| 15 | rs142215530 | A | G | 0.13 | 0.04 | 0.04 | 0.11 | 0.03 | 0.13 |
| 15 | rs11637565 | G | A | 0.64 | 0.78 | 0.70 | 0.61 | 0.68 | 0.54 |
| 16 | rs391855 | A | T | 0.38 | 0.81 | 0.73 | 0.43 | 0.70 | 0.40 |
| 16 | rs305065 | G | C | 0.66 | 0.99 | 0.92 | 0.65 | 0.91 | 0.69 |
| 18 | rs1036935 | A | G | 0.79 | 0.63 | 0.68 | 0.79 | 0.62 | 0.73 |
| 18 | rs4368253 | T | C | 0.68 | 0.72 | 0.64 | 0.68 | 0.72 | 0.72 |
| 18 | rs77551289 | A | G | 0.13 | 0.00 | 0.02 | 0.10 | 0.02 | 0.07 |
| 18 | rs4987852 | T | C | 0.07 | 0.00 | 0.01 | 0.07 | 0.01 | 0.10 |
| 19 | rs7254272 | G | A | 0.19 | 0.74 | 0.53 | 0.16 | 0.58 | 0.17 |
| 19 | rs874460 | C | A | 0.13 | 0.02 | 0.08 | 0.15 | 0.05 | 0.11 |
| 22 | rs140522 | T | C | 0.68 | 0.39 | 0.45 | 0.68 | 0.49 | 0.65 |

EA, European Ancestry; AA, African American; AAF, alternate allele frequency

**Supplemental Figure 1: Flow Chart of Study Population**

**
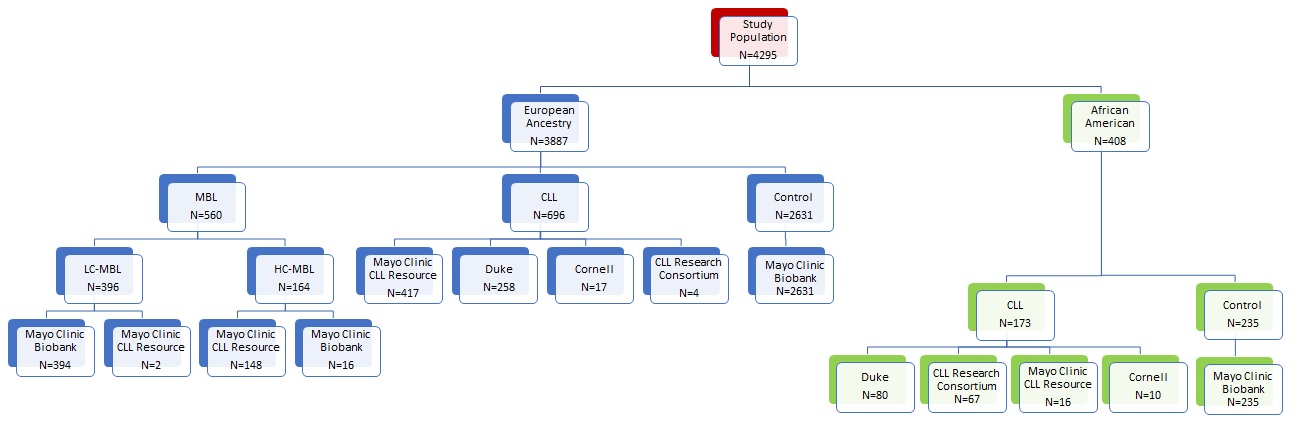
**

**Supplemental Figure 2: Scatter plot of 41 CLL risk SNPs by MAF of African American and European Ancestry**


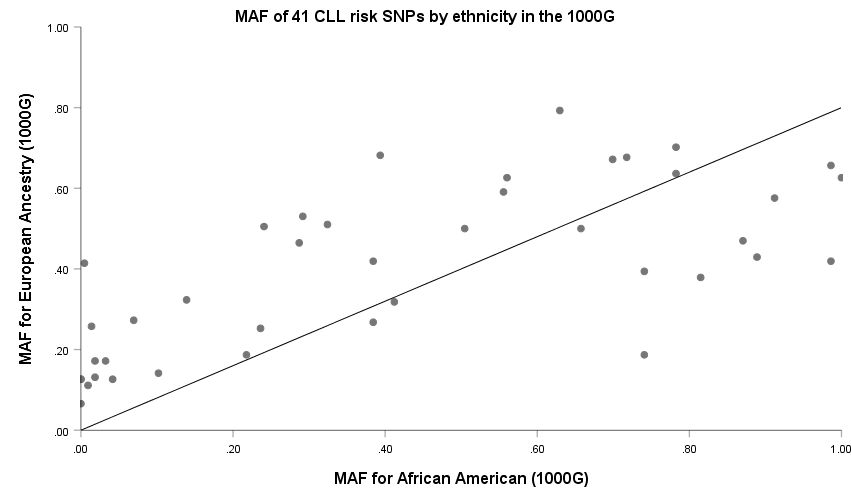


CLL, chronic lymphocytic leukemia; SNPs, single nucleotide polymorphism; MFA, minor allele frequency

**Supplemental Figure 3: Scatter plot for odds ratios of 41 CLL risk SNPs of CLL cases versus controls by ethnicity**


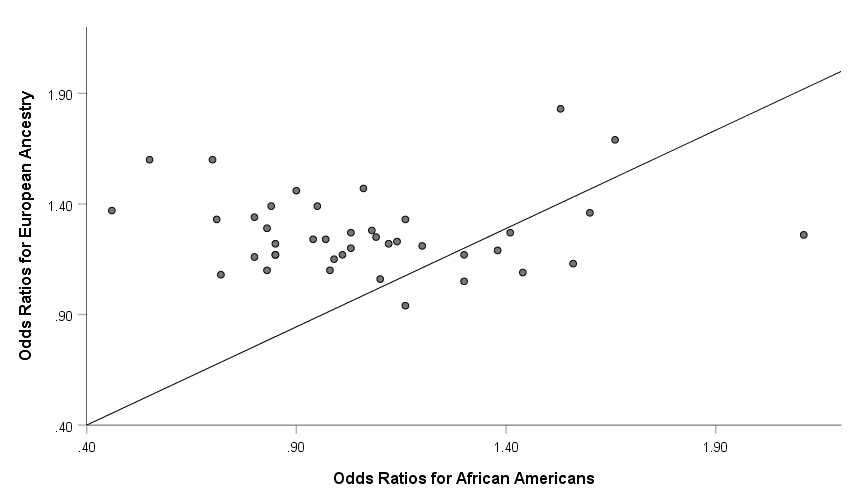


CLL, chronic lymphocytic leukemia; SNPs, single nucleotide polymorphism

**Supplemental Figure 4:** Polygenic risk score distribution by the Mayo Clinic Biobank controls and controls from prior studies (InterLymph)


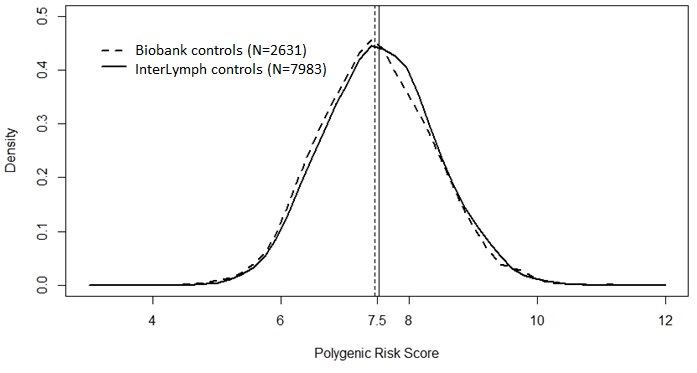


Histograms of polygenic risk scores (x-axis) and density (y-axis) by the InterLymph controls from our prior studies (solid black line) and the Mayo Clinic Biobank controls (dashed black line). InterLymph, International Lymphoma Epidemiology Consortium

References:

1. Law PJ, Berndt SI, Speedy HE, et al. Genome-wide association analysis implicates dysregulation of immunity genes in chronic lymphocytic leukaemia. *Nature Communications*. 2017;8:14175. doi:10.1038/ncomms14175

2. Kleinstern G, Camp NJ, Goldin LR, et al. Association of polygenic risk score with the risk of chronic lymphocytic leukemia and monoclonal B-cell lymphocytosis. *Blood*. 2018;131(23):2541-2551. doi:10.1182/blood-2017-11-814608
